# Liver transplantation in glycogen storage disease: a single-center experience

**DOI:** 10.1186/s13023-022-02284-y

**Published:** 2022-03-21

**Authors:** Zahra Beyzaei, Alireza Shamsaeefar, Kurosh Kazemi, Saman Nikeghbalian, Ali Bahador, Masoud Dehghani, Seyed-Ali Malekhosseini, Bita Geramizadeh

**Affiliations:** 1 Shiraz Transplant Research Center (STRC), Shiraz University of Medical Sciences, Khalili St., Research Tower, Seventh Floor, Shiraz, Iran; 2 Department of Hepatobiliary Surgery, Abu-Ali-Sina Hospital, Shiraz University of Medical Sciences, Shiraz, Iran; 3 Department of Pathology, Medical School of Shiraz University, Shiraz University of Medical Sciences, Shiraz, Iran

**Keywords:** Glycogen storage disease, Liver transplantation, Outcome, Cirrhosis, Metabolic control

## Abstract

**Background:**

Glycogen storage diseases (GSDs) are inherited glycogen metabolic disorders which have various subtypes. GSDs of type I, III, IV, VI, and IX show liver involvement and are considered as hepatic types of GSDs. Thus, liver transplantation (LT) has been proposed as a final therapy for these types of GSD. LT corrects the primary hepatic enzyme defect; however, the long-term outcomes of LT in these patients have not been extensively evaluated so far. There are few reports in the English literature about the outcome of GSD patients after LT. There has been no report from Iran. The present retrospective study aimed to evaluate the long-term outcomes of eight patients with GSD types I, III, and IV who underwent LT in the affiliated hospitals of Shiraz University of Medical Sciences, from March 2013 to June 2021. During this period, there were no patients with GSD VI and IX identified in this center.

**Results:**

The median time of diagnosis of the GSDs and at transplant was 1 year and 11 years, respectively. All eight transplanted patients were alive at the time of follow-up in this study. None of them required a re-transplant. All of the patients showed normalized liver enzymes after LT with no sign of hypoglycemia.

**Conclusions:**

LT is an achievable treatment for end-stage hepatic involvement of GSDs with a cure for metabolic deficiency. Our experience in these eight patients shows a favorable outcome with no mortality and no major complication.

## Background

Glycogen storage diseases (GSDs) refer to a group of inherited disorders caused by the absence of essential enzymes in the synthesis or degradation of glycogen [1, 2]. The most significant and severe liver dysfunction is observed in GSD types I, III, and IV [3]. The common presentations of these patients include hypoglycemia, hepatomegaly, and lactic acidosis.

Prevention of hypoglycemia has a key role in the treatment of patients with hepatic GSD except for GSD IV, in which hypoglycemia is a late feature of hepatic failure. Patients with these types of GSD can be maintained normoglycemic by taking cornstarch [4]. If good metabolic control is not achieved by dietary interventions and medical management, liver transplantation (LT) is considered as the curative treatment. Progressive liver failure, liver cirrhosis and the risk of hepatocellular carcinoma are still other indications that make patients with GSD potential candidates for LT. LT can preserve normal growth in the pediatric population by improving liver enzyme defects and metabolic abnormalities [5]. Nonetheless, few studies have been conducted on the outcomes of LT in GSD subtypes. Some reports on LT in pediatric patients with GSD I have shown excellent outcomes after a long-term follow-up [6].

There has not been any publication about GSD patients and liver transplantation from Iran. Therefore, our study aimed to investigate and evaluate the long-term and post-liver transplantation experience of patients with GSD. In this context, we describe our experience with the long-term outcome of eight patients with GSD who underwent LT during the last 10 years. It is worthy to note that our center is the only pediatric LT in Iran.

### Methods

We searched our institutional patient database to identify the records of patients with GSD who underwent a liver transplant at the affiliated hospitals of Shiraz University of Medical Sciences, between March 1, 2013, and June 31, 2021. All patients’ demographics, clinical features, biochemical investigations, histopathological results, and diagnostic imaging related to pretransplant assessment, transplant details, and post-transplant complications were retrospectively collected via the electronic and paper charts of patients. Demographic and perioperative characteristics were summarized using descriptive statistics. Continuous data were presented as the mean and standard deviation (SD) or median and range using SPSS 26.0 for Windows (SPSS Inc., Chicago, IL, USA). This study protocol conformed to the ethical guidelines of the 1975 Declaration of Helsinki as approved by Shiraz University of Medical Sciences.

## Results

### Preoperative clinical characteristics

A retrospective chart review was performed on eight patients (one girl and seven boys) with GSD who underwent LT at the affiliated hospitals of Shiraz University of Medical Sciences in 8 years. Three patients had GSD Ia, three most likely had GSD III and two patients most likely had GSD IV. All of the patients presented with hepatomegaly, hypoglycemia, as well as growth and developmental delay during childhood. For all patients, liver biopsies were performed with histopathological features and confirmed hepatic involvement of GSD (glycogen storage). The histologic findings were quite characteristic of GSD; there were hepatocytes with clear cytoplasm and intracytoplasmic glycogen accumulation. All of the eight cases showed severe fibrosis and micronodules of cirrhosis. For four patients, the GSD types were confirmed via DNA sequencing (P1, P3, P6, P7). Subsequent diagnostic analysis was also performed including related investigations such as full hematology and biochemistry tests as well as imaging studies. All patients transplanted because of progressive liver cirrhosis, and poor metabolic control (mean age: 14.75 years, range 3–38 years). All eight cases had elevated liver enzymes, hypertriglyceridemia, and hypercholesterolemia; also, one patient (P3) had hyperuricemia. Two patients experienced at least one episode of hypoglycemic seizure (P2, P5). Two patients (P1, P3) had platelet dysfunction and repeated epistaxis. Two patients (P7, P8) had pre-transplant coagulopathy which was managed with anticoagulant medication. The pre-transplant echocardiography was normal. In GSD Ia patients, there was not any evidence of proteinuria. All patients have conventionally started to take cornstarch before the age of two. Liver transplantation was performed late after the cirrhotic change had become established.

### Operative and donor characteristics

All patients underwent liver transplants by a standard protocol using the piggy-back technique [7]. No intraoperative complications were observed among these eight patients. None of them had hepatocellular carcinoma in the explanted livers. The main post-LT immunosuppressive protocol for all the patients was Cyclosporine/Sirolimus, Cellcept, and steroids (prednisolone). Five patients received grafts from deceased donors, while three patients received grafts from living-related donors. None of them needed a second liver transplant or combined organ transplant. All transplants were ABO-compatible. Two patients (P2, P7) were hospitalized several times due to several infectious episodes after the successful LT, including oral, respiratory, and renal infections and otitis which appropriate treatment and antibiotics were prescribed. The transplant information and outcomes following LT are summarized in Table 1.

**Table 1:** Transplant-related data

Patient	Gender	Type of GSD	Age at diagnosis (y)	Age at transplant (y)	Indication for transplant	Donor	Donor age	Blood type combination	Graft type	Mutation variant	Follow-up (y)	Pre-transplant liver biopsy	Post-transplant liver biopsy	Outcome
P1	Male	GSD Ia	1	16	Cirrhosis	DD	38	Compatible	Whole graft	p.G188R G641 → C	7.8	Macrovesicular steatosis with hepatitis, clear cytoplasm, severe fibrosis	Normal	Good graft function
P2	Male	Most likely GSD IV	0.5	3	Failure to thrive, Cirrhosis	LD (Mother)	35	Compatible	Left lateral segment	–	7.7	Intracytoplasmic inclusion, severe fibrosis	Mild gross hepatomegaly, microvesicular steatosis	Good graft function
P3	Male	GSD Ia	0.83	21	Cirrhosis	DD	15	Identical	Whole graft	c.79delC(p.Q27Xfs)	7.5	Macrovesicular steatosis with hepatitis, clear cytoplasm, severe fibrosis	Normal	Good graft function
P4	Male	Most likely GSD III	2	38	Increasing liver size and Cirrhosis	DD	19	Identical	Whole graft	–	7.4	Plant-like hepatocyte with clear cytoplasm, severe fibrosis	Normal	Good graft function
P5	Male	Most likely GSD III	1.5	3	Failure to thrive and Cirrhosis	LD (Father)	36	Identical	Left lateral segment	–	7.4	Plant-like hepatocyte with clear cytoplasm, severe fibrosis	Mild gross hepatomegaly, microvesicular steatosis	Good graft function
P6	Male	GSD Ia	0.7	6	Cirrhosis	DD	4	Identical	Whole graft	c.70 C > T(p.Q24X)	5.5	Macrovesicular steatosis with hepatitis, clear cytoplasm, severe fibrosis	Normal	Good graft function
P7	Male	GSD IV	1	3	Failure to thrive and Cirrhosis	LD (Mother)	29	Identical	Left lateral segment	c.998 A > T (p.Glu333Val),c.292G > C(p.Val98Leu)	2.8	Intracytoplasmic inclusion, severe fibrosis	Mild gross hepatomegaly, microvesicular steatosis	Good graft function
P8	Female	Most likely GSD III	1.5	28	Increasing liver size and Cirrhosis	DD	35	Identical	Whole graft	–	0.8	Plant-like hepatocyte with clear cytoplasm, severe fibrosis	Normal	Good graft function

*DD* deceased donor, *LD* living donor

### Postoperative results: correction of biochemical abnormalities

Following LT, the biochemical abnormalities improved considerably and no further hypoglycemic episodes occurred except for one patient (P5). All patients reverted to a normal diet after LT.

The hypertriglyceridemia and hypercholesterolemia were gradually corrected in 7 patients after 6–12 months postoperatively. However, patient 5 still had borderline hyperlipidemia because of his high protein and lipid diet which was referred to a nutritionist. Also, in P8, hyperlipidemia was observed either in short follow-up or her immunosuppressive protocol involving the use of steroids.

In P3, hyperuricemia improved after liver transplantation with normalized liver function. In one of the patients (P5) hyperuricemia was seen after liver transplantation (started after 2 years) which was most probably drug-induced side effects secondary to tacrolimus. The liver enzymes were normalized in all patients after 6 months post-transplantation. It should be mentioned that in the last follow-up, P5, P6, and P7 had COVID-19 infection and elevated alkaline phosphatase levels to 565, 662, and 1750 U/L respectively, which was normalized after recovery. Besides, one patient (P4) had persistent hypertension 6 months after transplantation which was treated with antihypertensive drugs. Also, biventricular hypertrophy with abnormal systolic and diastolic ventricular function by echocardiography was observed in this patient. Histopathologic evaluation after transplantation revealed small droplet macrovesicular steatosis in three patients, while it was normal in the other patients (Table 1). No mass was identified. Also, the hilar structures were unremarkable. Interestingly, our data showed that P1 had post-transplant coagulopathy, and the INR is now 2.0. His coagulopathy was successfully managed by proper medication. Ultrasound of the kidneys was done for all patients, which showed evidence of stones without any signs of hydronephrosis or solid cystic lesions. No renal impairment in GSD type I patients (P1, P3, P6) was observed. A renal biopsy was not performed. All GSD I patients were followed for at least 5 years and have not presented any sign of renal disease so far. All data are presented in Table 2.

**Table 2:** Pre- and post-transplant laboratory data

LAB findings	P1		P2		P3		P4		P5		P6		P7		P8	
	Pre-LT	Post-LT	Pre-LT	Post-LT	Pre-LT	Post-LT	Pre-LT	Post-LT	Pre-LT	Post-LT	Pre-LT	Post-LT	Pre-LT	Post-LT	Pre-LT	Post-LT
FBS^a^	45	90	82	90	47	86	60	86	59	90	45	79	75	90	55	86
TG^b^ (mg/dL)	210	82	131	77	235	89	219	70	105	168	332	50	124	91	234	198
T. Chol^b^ (mg/dL)	146	122	114	49	189	125	143	110	144	141	121	176	145	139	99	96
LDL^b^ (mg/dL)	33.6	67	65	25.6	93	65	87	25	–	80	–	103	–	60	–	47
HDL^b^ (mg/dL)	24	39	43	8	49	42	17	121	–	29	–	49	–	51	–	28
PT	13	19.6	20	13.3	14.7	13	13.3	13	15.3	15.8	23	13.2	29.2	12	25.4	13
INR	1.01	2.01	2.0	1.11	1.24	1	1.04	1	1.32	1.18	2.67	1.09	3.03	1	2.09	1.32
ALT^c^ (U/L)	128	35	118	11	143	21	74	32	85	29	51	20	64	90	71	20
AST^c^ (U/L)	117	38	261	40	183	19	80	37	53	31	110	33	199	60	94	16
ALK P^c^	229	135	397	115	658	121	374	153	221	201	820	229	622	117	348	148
BUN^d^	20	17	19	6	10	48	15	37	28	13	13	14	12	23	8	19
Cr^e^	0.1	1	0.3	0.1	0.6	1.1	0.3	0.6	0.4	0.9	0.6	0.8	0.3	0.5	0.5	0.8
Alb^f^	4.2	4.2	2.8	2.6	3.7	4.5	3.8	4.2	3.5	3.8	4.1	4.5	2.1	4.1	3.4	4
Uric acid^g^ (mg/dl)	5.9	3	3.8	2.1	10.5	4.6	–	4.5	7.9	10.3	7.9	3.9	3.9	4.1	–	–

^a^Reference range for FBS: normal 60–100 mg/dL; fasting > 60 mg/dL

^b^Reference range for TG, total cholesterol: < 150 mg/dL; LDL: 100–129; HDL: 45 mg/dL or higher

^c^Reference range for liver enzymes: ALT: < 40 U/L; AST: < 45 U/L; ALK P: 64–306 for females and 80–306 for males

^d^Reference range for BUN: 6–21 mg

^e^Reference range for creatinine: 8.0–1.3 for males and 0.6–1.3 mg for females

^f^Reference normal range for Alb: 3.5–5.4 g/dL

^g^Reference normal range for uric acid: 3.5–8.2 mg/dL

### Long-term outcomes

All recipients are currently alive with a good liver graft, muscle, and renal function at a median follow-up of 6 years and 4 months (follow up range 0.8–8.3 years after LT). No patient suffered from acute rejection, chronic allograft rejection, and post-transplant lymphoproliferative disorder (PTLD) during the follow-up. The results also showed good prognosis of liver transplantation in the patients with the diagnosis of GSD with more than 6 years survival rate.

## Discussion

LT is a well-established procedure for the treatment of patients with hepatic GSDs. It is done in patients with GSD I due to growth retardation, adenoma, or hepatocellular carcinoma, while patients with GSD III or IV frequently undergo LT for cirrhosis and complications of liver failure [8–10]. To the best of our knowledge, this is the first study on the outcomes of LT amongst patients with GSD types I, III, IV from Iran. The most common indication for LT in our patients was cirrhosis. Of note, none of the explanted liver histology showed malignant changes. In other reports, indications for LT included multiple hepatic adenomas or hepatocellular carcinoma [11]. Therefore, indications are different in GSD patients for LT which are particularly acceptable in cases where optimal metabolic management has not been achieved [12, 13].

As for metabolic control, previous reports have shown the correction of metabolic abnormalities and acceleration in development and growth in pediatric patients with GSD after LT [6]. It is mostly a major concern for patients with GSD IIIa, and IV who may present extrahepatic manifestations such as the muscle, heart, or nervous system. Therefore, the accumulation of glycogen in extrahepatic organs might be a potential risk for these patients. None of our patients with hypoglycemia experienced recurrent hypoglycemia after LT, which is similar to other studies [4–6]. Concerning GSD type IV, our results demonstrated that LT normalized the liver function of patients and improved the metabolic outcome. Thus, it appears that LT is an efficient treatment available for the classic type of GSD IV due to cirrhosis and progressive liver disease [14]. However, further follow-up is needed for the assessment of other complications such as skeletal myopathy, cardiomyopathy, and neuromuscular disorders.

Of note, hepatocyte transplantation and gene therapy are less invasive approaches for application in both preclinical and clinical studies. Although the long-term follow-up of hepatocyte transplantation has not been reported, short-term outcomes have been promising in clinical studies [15]. Gene therapy has efficaciously corrected the glycogen storage of GSD I as well as a promising approach regarding the potential of gene therapy to treat GSDs type III, IV [16].

Our results in the LT of GSD patients revealed excellent long-term post-liver transplant survival which is similar to developed countries from North America and Europe [4–6, 9, 17, 18]. Compared with other causes of LT in our center, GSD patients’ survival was better than other metabolic disorders such as Tyrosinemia type 1 and progressive familial intrahepatic cholestasis (PFIC) [19, 20]. For example, the 5-year survival of the patients with PFIC after LT was reported as 60% from our center [20]. In other metabolic studies, the mortality rate after transplantation has been higher than GSD patients [21, 22]. It should be also noted that the experience of LT in children with GSDs remains extremely limited, so pediatric patients with GSD must always be managed with consideration for the optimal timing of LT [23]. Thus, advances in immunosuppression protocols and surgical techniques may strengthen the case for early consideration of LT in GSD pediatric patients, which may improve the survival rate of patients after LT.

This cohort had some limitations such as retrospective study and a small number of patients. Another limitation was the absence of any patient with GSD VI and IX in this period in our center. Although this long-term observational study showed that LT was an appropriate, safe procedure for the late stage of liver involvement, for confirmation of this conclusion, a further large-scale study should be conducted.

In conclusion, all GSD patients in our center have shown acceptable post-LT outcomes on long-term follow-up. They represented improved metabolic control, liver function, and normal fasting tolerance after LT which improved the quality of life of these patients. Although some complications such as poor growth or sepsis might be caused by disease progression, most seemed related to immune suppression. Therefore, LT is a feasible option, with acceptable outcomes and good long-term results for GSD patients after failed medical treatment.

## Data Availability

The data that support the findings of this study are available from the corresponding author upon reasonable request.

## Abbreviations

GSD: Glycogen storage disease
IS: Immune suppression
LT: Liver transplant
PFIC: Progressive familial intrahepatic cholestasis
PTLD: Post-transplant lymphoproliferative disorder

## References

[1] Chen Y, Scriver CRBA, Sly WS, Valle D (2001). Glycogen storage diseases. The metabolic and molecular bases of inherited disease.

[2] Beyzaei Z, Geramizadeh B (2019). Molecular diagnosis of glycogen storage disease type I: a review. EXCLI J.

[3] Davis MK, Weinstein DA (2008). Liver transplantation in children with glycogen storage disease: controversies and evaluation of the risk/benefit of this procedure. Pediatr Transpl.

[4] Yuen YW, Quak SH, Aw MM, Karthik SV (2021). Long-term outcome after liver transplantation in children with type 1 glycogen storage disease. Pediatr Transpl.

[5] Iyer SG, Chen CL, Wang CC, Wang SH, Concejero AM, Liu YW, Yang CH, Yong CC, Jawan B, Cheng YF, Eng HL (2007). Long-term results of living donor liver transplantation for glycogen storage disorders in children. Liver Transpl.

[6] Shimizu S, Sakamoto S, Horikawa R, Fukuda A, Uchida H, Takeda M (2020). Long-term outcomes of living donor liver transplantation for glycogen storage disease type 1b. Liver Transpl.

[7] Tzakis A, Todo S, Starzl TE (1989). Orthotopic liver transplantation with preservation of the inferior vena cava. Ann Surg.

[8] Beyzaei Z, Ezgu F, Geramizadeh B, Imanieh MH, Haghighat M, Honar N (2021). Clinical and genetic spectrum of glycogen storage disease in Iranian population using targeted gene sequencing. Sci Rep.

[9] Liu PP, De Villa V, Chen YS, Wang CC, Wang SH, Chiang YC (2003). Outcome of living donor liver transplantation for glycogen storage disease. Transpl Proc.

[10] Beyzaei Z, Geramizadeh B, Karimzadeh S (2020). Diagnosis of hepatic glycogen storage disease patients with overlapping clinical symptoms by massively parallel sequencing: a systematic review of literature. Orphanet J Rare Dis.

[11] Boers S, Visser G, Smit P, Fuchs S (2014). Liver transplantation in glycogen storage disease type I. Orphanet J Rare Dis.

[12] Squires JE (2020). When considering liver transplant for children with glycogen storage disease 1b. Liver Transpl.

[13] Choi Y, Yi NJ, Ko JS, Moon JS, Suh SW, Lee JM, Jeong JH, Kim H, Lee HW, Lee KW, Suh KS (2016). Reappraisal of the role of portacaval shunting in the growth of patients with glycogen storage disease type I in the era of liver transplantation. Transplantation.

[14] Liu M, Sun LY (2021). Liver transplantation for glycogen storage disease type IV. Front Pediatr.

[15] Lee KW, Lee JH, Shin SW, Kim SJ, Joh JW, Lee DH (2007). Hepatocyte transplantation for glycogen storage disease type Ib. Cell Transpl.

[16] Kishnani PS, Sun B, Koeberl DD (2019). Gene therapy for glycogen storage diseases. Hum Mol Genet.

[17] Matern D, Starzl TE, Arnaout W, Barnard J, Bynon JS, Dhawan A, Emond J, Haagsma EB, Hug G, Lachaux A, Smit GP, Chen YT (1999). Liver transplantation for glycogen storage disease types I, III, and IV. Eur J Pediatr.

[18] Maheshwari A, Rankin R, Segev DL, Thuluvath PJ (2012). Outcomes of liver transplantation for glycogen storage disease: a matched-control study and a review of literature. Clin Transpl.

[19] Bahador A, Dehghani SM, Geramizadeh B, Nikeghbalian S, Bahador M, Malekhosseini SA, Kazemi K, Salahi H (2014). Liver transplant for children with hepatocellular carcinoma and hereditary tyrosinemia type 1. Exp Clin Transplant.

[20] Geramizadeh B, Mardani Z, Shojazadeh AR, Shamsaeefar AR, Kazemi K, Dehghani M, Malekhosseini SA. Liver transplantation in progressive familial intrahepatic cholestasis, a single center experience. IJTOM 2021.

[21] Morioka D, Kasahara M, Takada Y (2005). Living donor liver transplantation for pediatric patients with inheritable metabolic disorders. Am J Transplant.

[22] Karaca CA, Yilmaz C, Farajov R, Iakobadze Z, Aydogdu S, Kilic M (2019). Live donor liver transplantation for type 1 tyrosinemia: an analysis of 15 patients. Pediatr Transpl.

[23] Kasahara M, Horikawa R, Sakamoto S, Shigeta T, Tanaka H, Fukuda A, Abe K, Yoshii K, Naiki Y, Kosaki R, Nakagawa A (2009). Living donor liver transplantation for glycogen storage disease type Ib. Liver Transpl.

